# The phenotypic signature of adaptation to thermal stress in *Escherichia coli*

**DOI:** 10.1186/s12862-015-0457-3

**Published:** 2015-09-02

**Authors:** Shaun M. Hug, Brandon S. Gaut

**Affiliations:** Department of Ecology and Evolutionary Biology, UC Irvine, 321 Steinhaus Hall, Irvine, CA 92697 USA

## Abstract

**Background:**

In the short-term, organisms acclimate to stress through phenotypic plasticity, but in the longer term they adapt to stress genetically. The mutations that accrue during adaptation may contribute to completely novel phenotypes, or they may instead act to restore the phenotype from a stressed to a pre-stress condition. To better understand the influence of evolution on the diversity and direction of phenotypic change, we used Biolog microarrays to assay 94 phenotypes of 115 *Escherichia coli* clones that had adapted to high temperature (42.2 °C). We also assayed these same phenotypes in the clones’ ancestor under non-stress (37.0 °C) and stress (42.2 °C) conditions. We explored associations between Biolog phenotypes and genotypes, and we also investigated phenotypic differences between clones that have one of two adaptive genetic trajectories: one that is typified by mutations in the RNA polymerase β-subunit (*rpoB*) and another that is defined by mutations in the *rho* termination factor.

**Results:**

Most (54 %) phenotypic variation was restorative, shifting the phenotype from the acclimated state back toward the unstressed state. Novel phenotypes were more rare, comprising between 5 and 18 % of informative phenotypic variation. Phenotypic variation associated statistically with genetic variation, demonstrating a genetic basis for phenotypic change. Finally, clones with *rpoB* mutations differed in phenotype from those with *rho* mutations, largely due to differences in chemical sensitivity.

**Conclusions:**

Our results contribute to previous observations showing that a major component of adaptation in microbial evolution experiments is toward restoration to the unstressed state. In addition, we found that a large deletion strongly affected phenotypic variation. Finally, we demonstrated that the two genetic trajectories leading to thermal adaptation encompass different phenotypes.

**Electronic supplementary material:**

The online version of this article (doi:10.1186/s12862-015-0457-3) contains supplementary material, which is available to authorized users.

## Background

**Fig. 1:**
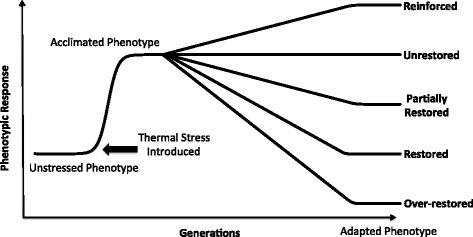
Schematic of acclimation and the potential directional outcomes of adaptation. In addition to restored and unrestored states, which reflect the phenotype of the unstressed and stressed ancestor, respectively, evolved clones may exhibit partially restored, over-restored or reinforced phenotypes. Not shown are cases of novelty, in which evolved clones differ from ancestral treatments that do not differ

Our understanding of the dynamics of adaptation in populations is incomplete [1], particularly with respect to the repeatability and the direction of adaptation. For repeatability, the major questions are, first, whether replicated evolutionary events converge on a single adaptive phenotype and, second, whether convergent phenotypes are caused by the same set of underlying genetic changes. For the direction of adaptation, the major question is whether adaptation commonly leads to novel phenotypes or instead acts to restore phenotypes to pre-stress states. To understand this last point, it is important to recognize that adaptation often begins with a physiological stress in a new environment. In the short term, there may be acclimation to stress through a physiological response, but genetic and phenotypic adaptation occurs in the longer term. The question is whether adaptation typically restores phenotypes to a pre-stress state or more often leads to phenotypic novelty (Fig. 1).

Questions about novelty, restoration, and convergence have been addressed in the context of experimental evolution [2–4]. These studies have found that evolution typically proceeds toward the restoration of the pre-stress condition. For example, Carroll and Marx [3] evolved eight replicate bacterial lineages under stress conditions and then measured gene expression. They found that 93 % of all adaptive changes in gene expression restored expression from the acclimated (stressed) state back to the wild-type (pre-stress) condition. Of these restorative changes, 70 % occurred in parallel across all eight populations. These studies make the important point that characterizing the intermediate acclimation process is essential to understanding the repeatability and direction of adaptation. However, these studies have also been limited to a low number (<10) of experimental replicates.

We recently performed a highly replicated experiment in which *Escherichia coli* evolved to high temperature [5]. To begin, we inoculated a clone of *E. coli* strain REL1206 [6] into 115 replicate populations, and then allowed the populations to evolve independently for 2000 generations at 42.2 °C. At the end of the experiment, we sequenced the genomes of single clones from each of the replicates. Our sequencing efforts revealed a total of 1331 mutations across a set of 115 evolved clones. Roughly half of these mutations were shared among two or more clones, with many falling into one of two different adaptive genetic trajectories. The first of these includes mutations in *rpoB*, which codes for the β-subunit of RNA polymerase, along with associated mutations in other RNA polymerase subunits and the *rod* genes that define cell shape. The second trajectory includes mutations in *rho*, which codes for a major transcription termination factor, along with mutations in *iclR*, a transcriptional regulator of the glyoxylate shunt of the Krebs cycle, and *cls*, a cardiolipin synthase gene important for regulating membrane fluidity and permeability. Mutations in the *rpoB* and *rho* adaptive trajectories are not mutually exclusive, but they are strongly negatively associated, presumably due to negative epistatic interactions [5].

Overall, we have observed adaptive genetic convergence—i.e., mutations in two or more independent clones—in ~80 genes [5], of which the two adaptive trajectories represent only a subset. We have been left, then, with a large amount of unexplained genetic diversity that is presumed to be adaptive at 42.2 °C, hundreds more mutations that are unique to single evolved clones, and the observation that all 115 of our populations independently evolved the ability to persist in the same high-temperature environment. While our study has provided a description of the breadth of genetic change underpinning an adaptive response [5], the extent of phenotypic convergence remains unclear, as does the direction of phenotypic evolution.

In this study, we assess phenotypic diversity among our 115 evolved *E. coli* clones using high-throughput Biolog arrays. Biologs are 96-well plates that test metabolic phenotypes [7], including 71 carbon utilization assays and 23 chemical sensitivity assays. Biologs have been used to discover new links in microbial biochemical pathways [8], to associate genotypes with phenotypes [9, 10], to uncover a decoupling between genotypic and phenotypic diversity across *E. coli* strains [11], and to validate patterns of long-term phenotypic evolution in diverse groups of bacteria [12]. They have also been employed in an evolution experiment to characterize ecological dynamics and niche displacement in coevolving subpopulations of *E. coli* [13].

Using Biolog assays, we have measured 94 phenotypes for each of our 115 evolved clones at 42.2 °C and for their REL1206 ancestor at two treatment temperatures (37.0 and 42.2 °C). With this dataset of phenotypes, our study has four interacting objectives. The first is to assess phenotypic variation among clones based on a Biolog ‘fingerprint’. The second is to measure the direction of phenotypic adaptation in our 115 evolved clones relative to the stressed (42.2 °C) and non-stressed (37.0 °C) ancestor. Based on previous studies [2, 3], we hypothesize that many of the phenotypic changes restore phenotypes from the stressed toward the pre-stress state. The third objective is to assess whether phenotypic shifts have a genetic component—i.e., to ascertain that genetic adaptation has contributed to the observed phenotypic shifts rather than phenotypic plasticity. Finally, we contrast the two adaptive trajectories typified by *rho* and *rpoB* mutations. Do these two genetic trajectories vary in their resultant phenotypes?

## Methods

### Evolution to thermal stress

Our thermal stress experiment was reported elsewhere [5], but we cover the experimental design here for the sake of clarity. Following previously established methods [14], we inoculated 10 mL of LB media with a loop taken from a freezer stock of Arabinose minus *E. coli* (REL1206), and the culture was grown overnight at 37.0 °C. A 0.1 mL aliquot of this culture was plated onto an LB plate, again at 37.0 °C, and colonies from this plate were used to inoculate each of the 115 independent lines for the evolution experiment. That is, each line was started from a separate and single bacterial colony. The lines were first grown overnight at 37.0 °C in a 10 ml LB culture to aid recovery, and then transferred in a 1:100 dilution to 9.9 ml of Davis minimal medium supplemented with 25 mg/L glucose (DM25) and grown overnight at 37.0 °C. Thereafter, each culture was transferred daily into fresh media via 100-fold dilution and maintained in a shaking water bath at 42.2 °C. The experiment continued for 2000 generations after the application of thermal stress. The ancestral REL1206 clone had been propagated previously at 37.0 °C for 2000 generations in DM25 and was thus likely to be well adapted to the media such that temperature (42.2 °C) was the major stress.

At the end of the 2000 generation experiment, we isolated one clone from each of the 115 populations, and each genome was sequenced. The clones were also assessed for their fitness relative to the ancestor at 42.2 °C [5]; on average, the evolved clones were ~40 % more fit than the ancestor. For further details, including relative fitness values and a table of genotypes, please refer to [5].

### Biolog assays

We streaked each of the 115 clones from the *E. coli* thermal stress experiment from frozen glycerol stocks onto tetrazolium and arabinose (TA) agar plates and grew them for one day at 37.0 °C. Although the clones had evolved at 42.2 °C in DM25, it is common practice in thermal stress studies to allow clones to recover from freezing under less stressful conditions [14–16].

For each clone, we chose colonies to assay on a GEN III Biolog MicroPlate. To perform the assay, we followed the manufacturer’s protocol, which included: *i*) inoculating bacterial colonies into Inoculating Fluid A (Biolog) to a turbidity between 97 and 99 % transmittance, as determined by optical density (OD) at 600 nm on a Synergy H1 Hybrid Multi-Mode Microplate Reader (Biotek); *ii*) adding 100 μL of inoculum to each of the 96 wells of a Biolog plate; *iii*) incubating each plate for 22.25 h at 42.2 °C, and *iv*) developing the assay by measuring optical density (OD) at 590 nm. The OD at 590 nm measured the amount of reduced tetrazolium redox dye in each well, providing a quantitative measurement of respiratory activity in each well of the plate. We performed three Biolog assays for each of the 115 evolved clones, for a total of 115 × 3 = 345 plates.

We applied the same Biolog procedures to the REL1206 ancestor, but it was incubated at one of two different treatment temperatures: 37.0 and 42.2 °C. Moreover, for each temperature we performed two sets of three replicates, with the replicate sets performed on different days in order to incorporate potential ‘day effects’ into the experimental design. Thus, we performed 6 × 2 = 12 assays with the REL1206 ancestor.

Because REL1206 evolved at 37.0 °C in DM25 from a lab strain of *E. coli* B, we assumed that 37.0 °C represents a non-stress condition, while 42.2 °C was a stressful environment.

### Statistical and directional analysis of phenotypes

Each Biolog plate contained 94 assay wells (or ‘tests’) and two control wells. For each plate, the OD for each test was normalized to the OD of the appropriate control well. For example, the 71 tests that measure carbon utilization were normalized to a negative control lacking any added metabolic substrates, and the 23 tests that measure chemical sensitivity were normalized to a positive control lacking an inhibitor but permitting a baseline level of assay development. We term the normalized OD values as ‘phenotype values,’ or PVs.

We first examined Pearson correlation coefficients between all pairs of 94 Biolog tests, based on the average PV $\left(\overline{\mathrm{PV}}\right)$ for each clone. Because $\overline{\mathrm{PV}}\mathrm{s}$ were highly correlated among tests, we reduced the dimensionality of log-transformed PV data using principle components analysis (PCA). PCA analyses were based on the R [17] module prcomp, with the flags retx=TRUE, center=TRUE, and scale=TRUE. Thereafter, we considered only principal components with significant eigenvectors, as determined by the ‘random average under permutation’ metric of Peres-Neto et al. [18], which was based on 1000 permuted datasets. The significance of loadings was examined with the bootstrap eigenvector metric of Peres-Neto et al. [19], based on 1000 resamplings. Both metrics have been shown to be well behaved on a range of simulated datasets [18, 19].

For each retained component of the PCA, we compared the average of scores ${\overline{S}}_{\mathrm{x}}$ of each evolved clone *x* to the average scores of REL1206 at 37.0 °C $\left({\overline{S}}_{37{}^{\circ}\mathrm{C}}\right)$ and at 42.2 °C $\left({\overline{S}}_{42{}^{\circ}\mathrm{C}}\right)$. We used *t*-tests for these pairwise comparisons, under the null hypothesis that the REL1206 scores did not differ from those of an evolved clone. The *p-*value for individual *t-*tests were determined by an empirical null distribution, based on 10^5^ permutations. For each set of comparisons, the resultant *p*-values were adjusted using a false discover rate (FDR) of *q* < 0.01, based on the p.adjust module of *R*.

Within each principal component, we categorized the direction of phenotypic evolution for each evolved clone by comparing the magnitude and significance of pairwise comparisons among ${\overline{S}}_{37{}^{\circ}\mathrm{C}},{\overline{S}}_{42{}^{\circ}\mathrm{C}}$ and ${\overline{S}}_{\mathrm{x}}$. Following previous literature [3], we defined a total of six directional categories, which represent the phenotypic consequences of evolution for clone *x* (Table 1 and Fig. 1).

**Table 1:** Categorizations of phenotypic magnitude and direction

Category	Condition^a^	Number^b^
Partially Restored	$$ \begin{array}{l}{\overline{S}}_{37{}^{\circ}\mathrm{C}}<{\overline{S}}_{\mathrm{x}}<{\overline{S}}_{42{}^{\circ}\mathrm{C}}\\ {}{\overline{S}}_{37{}^{\circ}\mathrm{C}}>{\overline{S}}_{\mathrm{x}}>{\overline{S}}_{42{}^{\circ}\mathrm{C}}\end{array} $$	155
Reinforced	$$ \begin{array}{l}{\overline{S}}_{\mathrm{x}}<{\overline{S}}_{42{}^{\circ}\mathrm{C}}<{\overline{S}}_{37{}^{\circ}\mathrm{C}}\\ {}{\overline{S}}_{\mathrm{x}}>{\overline{S}}_{42{}^{\circ}\mathrm{C}}>{\overline{S}}_{37{}^{\circ}\mathrm{C}}\end{array} $$	79
Over-restored	$$ \begin{array}{l}{\overline{S}}_{\mathrm{x}}>{\overline{S}}_{37{}^{\circ}\mathrm{C}}>{\overline{S}}_{42{}^{\circ}\mathrm{C}}\\ {}{\overline{S}}_{\mathrm{x}}<{\overline{S}}_{37{}^{\circ}\mathrm{C}}<{\overline{S}}_{42{}^{\circ}\mathrm{C}}\end{array} $$	30
Unrestored	$$ \begin{array}{l}{\overline{S}}_{42{}^{\circ}\mathrm{C}}\cong {\overline{S}}_{\mathrm{x}}<{\overline{S}}_{37{}^{\circ}\mathrm{C}}\\ {}{\overline{S}}_{42{}^{\circ}\mathrm{C}}\cong {\overline{S}}_{\mathrm{x}}>{\overline{S}}_{37{}^{\circ}\mathrm{C}}\end{array} $$	240
Restored	$$ \begin{array}{l}{\overline{S}}_{37{}^{\circ}\mathrm{C}}\cong {\overline{S}}_{\mathrm{x}}<{\overline{S}}_{42{}^{\circ}\mathrm{C}}\\ {}{\overline{S}}_{37{}^{\circ}\mathrm{C}}\cong {\overline{S}}_{\mathrm{x}}>{\overline{S}}_{42{}^{\circ}\mathrm{C}}\end{array} $$	123
Novel	$$ \begin{array}{l}{\overline{S}}_{42{}^{\circ}\mathrm{C}}\cong {\overline{S}}_{37{}^{\circ}\mathrm{C}}>{\overline{S}}_{\mathrm{x}}\\ {}{\overline{S}}_{\mathrm{x}}<{\overline{S}}_{42{}^{\circ}\mathrm{C}}\cong {\overline{S}}_{37{}^{\circ}\mathrm{C}}\end{array} $$	63
Uninformative	${\overline{S}}_{42{}^{\circ}\mathrm{C}}\cong {\overline{S}}_{\mathrm{x}}\cong {\overline{S}}_{37{}^{\circ}\mathrm{C}}$	334
Inconsistent	All Remaining Relationships^c^	11

^a^Throughout the table, the symbol ‘≅’ reflects a comparison between two $\overline{S}$ values that are similar enough that they do not differ statistically by *t*-test; however, ‘>’ and ‘<’ refer to significantly different values.

^b^Of 1035 total comparisons (115 clones × 9 principal components)

^c^Mostly resulting from non-transitive pairwise significance

Hierarchical clustering of clones was based on ${\overline{S}}_{\mathrm{x}}$. Clustering utilized Euclidean distances based on$\overline{\mathrm{PV}}$values; an unweighted pair group method with arithmetic mean (UPGMA) [20] was implemented in MATLAB. Multivariate analysis of variance (MANOVA) was used to test for differences in phenotypes between pre-defined groups of clones. MANOVA analyses used PV data from each test and each replicate as dependent variables and the groups as independent variables, resulting in the model (PVs ~ groups). MANOVA was implemented in the R function manova, based on the Pillai test of significance.

### Associations between Phenotype and Genotype

To test for associations between phenotypes and genetic mutations, we grouped mutations found in our evolved clones into ‘mutational objects.’ These groupings arose by classifying mutations into three broad classes: genic, intergenic, and multigenic. Genic mutations included all point mutations, small indels, and IS insertions that affected a single gene, and we grouped these into one mutational object whose identifier was the name of the affected gene. For example, an evolved clone possessing a point mutation in the *cls* gene and another evolved clone possessing an IS insertion in the *cls* gene each received a single identifier, ‘cls,’ to describe their mutations. Intergenic mutations comprised point mutations, small indels, and IS insertions that fell in noncoding regions between two genes, and we split these into two objects, one associated with each neighboring gene. Lastly, multigenic mutations comprised deletions and insertions spanning two or more genes; we classified these as their own objects whose identifiers were not associated with any specific gene. All genotypic data were from Tenaillon et al. [5], which includes a supplementary table of genotypes. We grouped mutations into objects because most mutations discovered within the thermal stress experiment were found in only a single clone, and hence provided no basis for associating genotype with phenotype. These groupings likely increased statistical power but may have had an unintended trade-off in statistical power if there was allelic heterogeneity.

For each of the mutational objects present in two or more evolved clones, the PCA scores from each evolved clone were placed into one of two groups: those possessing the mutational object (the cases), or those lacking the mutational object (the controls). A *t*-test assuming equal variance was used to determine whether the cases and controls differed significantly in each of the nine principal components. Results were corrected to *q* < 0.01.

## Results

### Phenotypic space

To better understand phenotypic evolution during a previously published evolution experiment [5], we performed a total of 357 Biolog assays on 115 evolved clones and two ancestral treatments. Each assay included 94 discrete tests. After normalizing OD readings, we first calculated $\overline{\mathrm{PV}}$ values for each test and each clone and then measured Pearson pairwise correlations between these tests. Of 8836 (=94 × 94) pairwise comparisons between tests, 20.8 % (1836) were significantly correlated after sequential Bonferroni correction at α = 0.01 (Additional file 1: Figure S1). Given substantial correlation between tests, we reduced the complexity of PV data by PCA transformation into orthogonal components. The first component of the PCA represented 31 % of the variance (Additional file 2: Figure S2), and the eigenvector of the first nine components was significant [18]. Each of the first nine components had eigenvectors >2.0 and together explained 68.7 % of variation. We retained the first nine components for further analysis.

**Fig. 2:**
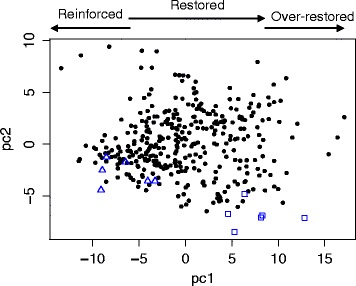
Plot of the first two principal components. The dots represent scores from the 115 evolved clones, each of which was replicated three times. The triangles represent the six replicates of the REL1206 ancestral strain at 42.2 °C; squares denote the ancestor at 37.0 °C. The arrows at the top of the plot illustrate directions of change relative to the two ancestral treatments (see Fig. 1 and Table 1)

Figure 2 plots the first and second principal components and helps convey two pieces of information about PCA scores. First, the ancestral data were typically well differentiated by treatment (37.0 or 42.2 °C). For example, the first component visually separated the sets of six ancestral replicates by treatment (Fig. 2). While the separation was less obvious for the second component, *t*-test comparisons between ${\overline{S}}_{37{}^{\circ}\mathrm{C}}$ and ${\overline{S}}_{42{}^{\circ}\mathrm{C}}$ indicated that the two ancestral treatments were significantly differentiated in seven of nine principal components (pc1, pc2, pc5, pc6, pc7, pc8 and pc9; *t*-test, unequal variances; sequential Bonferroni correction for α = 0.01). This differentiation represents the phenotypic effects of acclimation (Fig. 1).

A plot of the first two components also provides an opportunity to illustrate features of the direction and magnitude of evolutionary change (Fig. 2). In the first principal component, most score values clustered near the stressed (42.2 °C) ancestor, suggesting that most of the evolved clones were phenotypically unrestored in pc1. However, the scores of several clones fell intermediate between the two ancestral treatments or near the unstressed (37.0 °C) ancestor, indicating partial or full phenotypic restoration, respectively. We consider the direction of phenotypic change more formally below.

PCA also estimated loadings on each axis (Additional file 3: Table S1); these loadings provide information about individual tests that may have contributed variation to an axis. We tested for ‘significant’ loadings using a published bootstrapping heuristic, but none were significant at *p* < 0.05. The lack of significance reflects the fact that the loadings were fairly even among tests. For example, in pc1 the highest loading—in terms of the percent of the total loading values—was 2.0 % for metabolic activity on ‘glucuronamide,’ but altogether 31 of 94 tests contributed between 1.5 and 2 % to loadings in pc1. However, 27 of these 31 (87 %) were tests that measure OD on sugar substrates, suggesting that pc1 primarily reflects variation related to carbohydrate metabolism. The top loadings in pc2 were related primarily to tests either on Krebs cycle compounds or on amino acids, while the top loadings in pc3 included assays for chemical sensitivity (Additional file 3: Table S1).

### The direction of adaptation

The two ancestral treatments were significantly differentiated for seven of nine principal components. These observations lay the foundation for assessing the direction of evolution—i.e., did evolution tend to restore phenotypic traits to the non-stressed (37.0 °C) state, or did it lead to novel phenotypes?

To assess directionality more formally, we first tested for differences in ${\overline{S}}_{\mathrm{x}}$ between an evolved clone and each of the two ancestral treatments. For example, we compared ${\overline{S}}_{\mathrm{x}}$ of each of the 115 clones to ${\overline{S}}_{42{}^{\circ}\mathrm{C}}$ in each of the nine axes, for a total of 115 × 9 = 1035 contrasts, and found that 27.2 % (or 282 out of 1035) of tests were significant at *q* < 0.01. There were nonetheless more differences between the evolved clones and the 37.0 °C control, because 53.3 % (552 of 1035) of contrasts between ${\overline{S}}_{\mathrm{x}}$ and ${\overline{S}}_{37{}^{\circ}\mathrm{C}}$ were significant (*q* < 0.01). These results were similar to Fig. 2 in giving an overall impression that the evolved clones tended to be more similar in phenotype to the stressed ancestor than to the non-stressed ancestor.

We classified the results of *t*-tests into eight categories based on the direction and significance of comparisons among ${\overline{S}}_{\mathrm{x}},\kern0.5em {\overline{S}}_{37{}^{\circ}\mathrm{C}}$ and ${\overline{S}}_{42{}^{\circ}\mathrm{C}}$ (Table 1). Of 1035 comparisons, the highest number of tests (334 of 1035) fell into the ‘uninformative’ category, due to a lack of significance among comparisons. Among informative categories, the most comparisons were in the ‘unrestored’ (240) category, followed by ‘partially restored’ (155), ‘restored’ (123), ‘reinforced’ (79), ‘novel’ (63) and ‘over-restored’ (30) (Table 1).

**Fig. 3:**
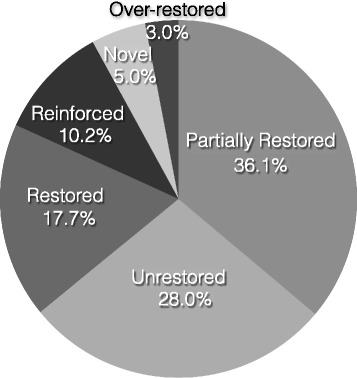
Pie chart reporting estimates of the proportion of phenotypic variation attributable to directions of adaptation

These categorical numbers reflect directionality, but they do not account for the fact that the nine principle components explained different proportions of variance (Additional file 2: Figure S2). To estimate the total proportion of variance explained by each directional category, we weighted results by the proportion of variance explained in each axis (Additional file 4: Table S2). Summing across all informative comparisons, weighting revealed that the biggest contributor to phenotypic variance was ‘partial restoration’ of the unstressed phenotype, which explained 36.1 % of observed phenotypic variation (Fig. 3). The category of ‘partial restoration’ was followed by the unrestored (28.0 %) and restored phenotypes (17.7 %). In contrast, novel, over-restored, and reinforced phenotypes combined to explain 18.2 % of variation.

### Phenotype-genotype associations

The predominant phenotypic response during our experiment was toward the partial or full restoration of the pre-stress condition. To verify that these shifts in phenotype had a genetic component—and therefore resulted from adaptive change rather than phenotypic plasticity—we used a case–control approach to associate scores with 165 mutational objects. In total, we found 117 significant (*q* < 0.01) associations with 70 mutational objects distributed across eight of the nine principal components (Table 2). We explored the validity of our phenotype-genotype associations by performing *t*-tests on random permutations of our case/control categories for each Biolog assay. When the absolute values of the *t*-statistics obtained from our observed genotypic groups were sorted and plotted against those obtained from randomized groups, there was a strong signal of more extreme *t*-statistics in the observed data (Additional file 2: Figure S3), suggesting a biological signal in our results.

**Table 2:** Significant (*q* < 0.01) associations between genetic and phenotypic variation

Principal component	Associated mutational objects (Number of affected clones)^a^
1	ECB_00503_large (35), ESCRE1901 (13), *trkD* (3), *dctA* (5), *yhjK* (5), *folM* (2), *yccE* (2), *metB* (2)
2	ECB_00503_large (35), hokE_large (3), *rpoB* (76), *rssB* (10), *yraL* (2), *rho* (45), *pta* (5), *fbp* (7), *cls* (56), oppD_large (3), *rpsR* (5), *ilvL* (30), *iclR *(37), *sdaC* (2), *kpsD* (13), *nth* (3)
3	*(none)*
4	*mrdA* (23), ECB_00503_large (35), *rpoB* (76), *fbaB* (26), *gatY* (26), *yhdJ *(2), *rho* (45), *lon* (4), *gmr* (2), *rnb* (2), *ygjF* (6), *cysH* (2), *fis* (5), *rpoH* (2), *ompR *(6), *pta* (5), *kpsE* (11), *metA* (4), ECB_00503_small (9)
5	*dnaG* (15), *omp* *F* (10),* rpoD* (36), *trkD* (3), *atoC* (2), *asnS* (8), hokE_large (3), *dctA* (5), *yhjK* (5), ESCRE1902 (3), *yebO* (3), *nusA* (12), IS150_insJ (4), ECB_00503_large (35), *yghD* (2), *yccE* (2), *mrdA* (23), *cls* (56)
6	*cls* (56),* rpoB* (76), *rho* (45), *glpF* (13), *mreB* (12), *rpsA* (3), ECB_00503_large (35), IS3_insF (3), *iclR* (37), *kpsM* (7), *pta* (5), *ygjF* (6), *kpsE* (11), *ycbC* (2), *nusA* (12)
7	*fbp* (7), *ompF* (10), *asnS* (8), *glpF* (13), *cysB* (6), *dctA* (5), *yhjK* (5), *dnaG* (15), cpsG_large (21), *dus* *B* (4), ECB_00503_large (35), *dnaA* (4), IS1_insB (2)
8	*fbp* (7), *rpsR* (5), *mreB* (12), *rho* (45), *folM* (2), *rssB* (10)
9	*dctA* (5), *yhjK* (5), *rho* (45), *uspA* (8),* rssB* (10), *cls* (56), *gltP* (7), *nrfG* (7),* iclR* (37), *glpT* (8), *kpsD* (13),* yifB* (25), *rhoL *(2), *ilvL* (30), ECB_01993 (2), ECB_01994 (2), IS186_insL (2), *ycbC* (2), *rpoB* (76), *ompF* (10), IS150_insK (2), *glyS* (2)

^a^Mutational objects are organized by *p*-value, in ascending order

Among the many significant genotype-phenotype associations (Table 2), a few were especially notable. For example, a large deletion variant (ECB_00503_large) that was common to 35 of the 115 evolved lines also had the lowest *p*-values in associations to the first two principle components, suggesting it had a major effect on adaptive phenotypes. Moreover, *rpoB* and *rho*, two genes that represent the two major adaptive trajectories [5], each exhibited significant associations to four and five principal components, respectively.

### Contrasting the *rho* and *rpoB* trajectories

Like previous experiments [2–4], we have shown that most phenotypic variation in our experiment was due to partial or full restoration of the unstressed phenotype. We have gone further to show that some of this variation associates with an underlying genetic component. However, we have not yet addressed the question as to whether different adaptive trajectories—particularly those that include *rho* and *rpoB*—differ in phenotype. We used two approaches to compare these two trajectories.

The first was to test for differences between clones with *rpoB* mutations and clones with *rho* mutations, using MANOVA applied directly to PV data. The results indicated that the two groups differ in phenotype (*p* < 2.2 × 10^−16^). MANOVA also assessed the significance of individual tests (or factors) between groups; 23 of the 94 Biolog assays differed significantly between the *rpoB* and *rho* groups at α = 0.01 (sequential Bonferroni correction) (Table 3). Among these 23 factors, the five with the lowest *p*-values were tests of chemical sensitivity.

**Table 3:** Individual Biolog tests that contribute significantly to differences between clones that contain *rpoB* vs. *rho* mutations

Biolog Test	*p*-value	Category
4 % NaCl	1.04E-25	Chemical Sensitivity
Sodium Butyrate	2.85E-22	Chemical Sensitivity
Lincomycin	1.61E-18	Chemical Sensitivity
Tetrazolium Blue	2.34E-14	Chemical Sensitivity
Nalidixic Acid	4.49E-10	Chemical Sensitivity
p-Hydroxy-Phenylacetic Acid	6.83E-10	Carboxylic Acids, Esters, Fatty Acids
Gelatin	1.53E-09	Amino Acids
D-Sorbitol	3.72E-08	Carbohydrates, Carbohydrate Derivatives
D-Salicin	9.24E-07	Carbohydrates, Carbohydrate Derivatives
Beta-Hydroxy-D,L-Butyric Acid	1.08E-06	Carboxylic Acids, Esters, Fatty Acids
Aztreonam	1.46E-06	Chemical Sensitivity
L-Arginine	1.57E-06	Amino Acids
D-Malic Acid	1.62E-06	Carboxylic Acids, Esters, Fatty Acids
Quinic Acid	1.90E-06	Carbohydrates, Carbohydrate Derivatives
L-Histidine	2.05E-06	Amino Acids
Inosine	3.17E-06	Carbohydrates, Carbohydrate Derivatives
L-Pyroglutamic Acid	5.92E-06	Amino Acids
N-Acetyl-Neuraminic Acid	6.21E-06	Carbohydrates, Carbohydrate Derivatives
pH 5	7.78E-06	Chemical Sensitivity
Tween 40	1.01E-05	Carboxylic Acids, Esters, Fatty Acids
D-Raffinose	2.70E-05	Carbohydrates, Carbohydrate Derivatives
Bromo-Succinic Acid	3.85E-05	Carboxylic Acids, Esters, Fatty Acids

**Fig. 4:**
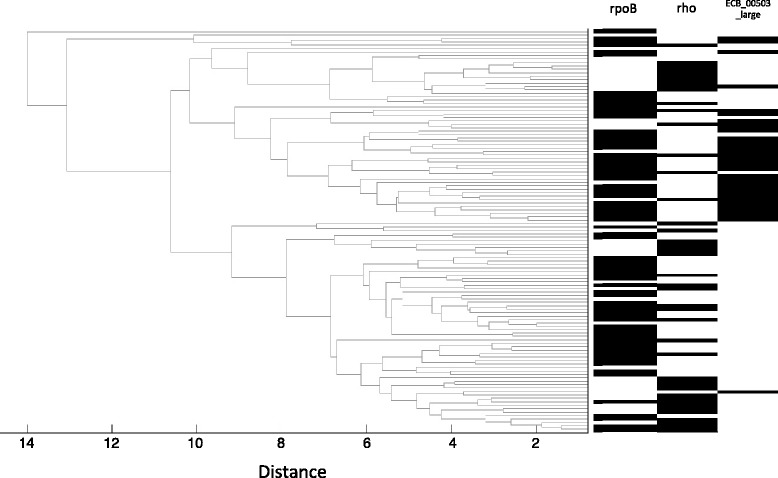
Hierarchical clustering of evolved lines by phenotypes. Dendrograms are labeled with the presence (black) or absence (white) of each of mutation in the *rho* gene, the *rpoB* gene and the large deletion (ECB_00503_large)

The second approach was hierarchical clustering of the 115 clones by phenotype, followed by visual examination of the distribution of *rho* and *rpoB* mutants on the dendrogram. We reasoned clones should group phenotypically according to genetic trajectory if the *rpoB* and *rho* trajectories lead to different Biolog phenotypes. The results were intriguing, if not completely clear (Fig. 4). The dendrogram showed that clones with either mutational object fell into clusters; that is, clones with *rpoB* mutations clustered into discrete groups, and likewise for clones bearing *rho* mutations. In addition, the clusters of *rho*- and *rpoB* clones tended to be mutually exclusive, as expected given that few clones carried mutations in both genes [5]. However, for each mutational object, there were multiple clusters, without a clear delineation between the two genetic groups.

## Discussion

Adaptation moves an organism toward a phenotypic optimum, but the question remains as to whether there is a single or several genetic trajectories to one or several optima. Previously, we evolved 115 separate *E. coli* lines under thermal stress (42.2 °C) for approximately one year (2000 generations), with the intent to measure the diversity of an adaptive response. This experiment revealed that each of the experimental lines improved in fitness by an average of 40 % across clones isolated from each line, fueled by the accumulation of ~11 mutations per clone on average [5]. The most frequently mutated genes were related to DNA transcription, particularly the *rpoB* and *rho* genes. Mutations within these two genes tended to be negatively associated.

It is an open question whether these two adaptive trajectories—or indeed, the >1200 mutations observed during the experiment—lead to convergent phenotypes beyond an increased ability to grow at 42.2 °C. Accordingly, this study has been designed to measure phenotypic diversity among these 115 *E. coli* clones using Biolog plates. These plates assess phenotypic characteristics by assaying metabolic activities and chemical sensitivities, but they have at least three important limitations. The first is that many of the phenotypes measured by Biolog plates may not have a direct relationship to fitness during the evolutionary experiment; they may represent pleiotropic effects. It is nonetheless an important task to characterize the phenotypic diversity generated during an adaptive response, as diversity may impact evolvability [21–23]. The second is that many of the assays are not independent (Additional file 1: Figure S1). The lack of independence necessitated orthogonal transformation of the data, but these transformations resulted in the loss of information and lessened the ability to associate a discrete phenotype (i.e., a specific Biolog test) to a causative genotype. Lastly, although Biolog technology measures utilization of carbon sources and resistance to inhibitors, bacterial growth and metabolism are complex and environment-dependent; as a result, changes in OD (or a lack thereof) are not always reliable indicators of bacterial growth and metabolism in each assay [24]. Nonetheless, changes in OD are consistent within our system, both across replicates and on different days, making the Biolog data a useful indicator of a phenotypic ‘fingerprint.’

### Restoration, not novelty, predominates in our experiment

Previous experiments have found that a major component of adaptation to a stressful state is the restoration of phenotypes to a non-stressed state [2–4]. Similar to these experiments, we find that the predominant phenotypic shift in our experiment was toward a restored state like that of the 37.0 °C ancestor. Together, full and partial restoration of phenotypes represent 53.8 % of the phenotypic variation among our evolved clones (Fig. 3).

In contrast, evolutionary novelty is less common, but the proportion of novel phenotypic variation varies by definition. Writ narrowly, novelty may be defined as an evolved state that differs from ancestral treatments that do not differ from each other (Table 1). Under this definition, novelty accounts for 5.0 % of phenotypic variation (Fig. 3). However, novelty can also be described more broadly as a phenotype beyond the limits of the two ancestral treatments, so that over-restoration and reinforcement also encompass novelty (Fig. 1). With this broader definition, novelty encompasses 18.2 % of variation but is still dwarfed by both partial and full restoration.

This general result—i.e., that novelty is a less frequent component of adaptation than restoration—is also consistent with previous studies. For example, Sandberg et al. [4] have found that just 13 % (101/804) of parallel gene expression shifts in their thermal stress experiment are reinforcements, a classification that can be considered a type of novelty in their system. Likewise, Carroll and Marx [3] have documented that cases of parallel novelty are rare in their gene expression data, occurring in just five out of thousands of genes.

Generalizing across studies, the predominant effect within microbial evolution experiments appears to be restoration, at least in the short term. As such, this directional response likely indicates pressure to compensate for the metabolic and energy requirements of the stress response. In the case of *E. coli* thermal stress, the immediate response to thermal stress—i.e., the heat shock response (HSR)—has been well characterized. The HSR up-regulates expression of the transcription factor σ^32^, thereby driving increased expression of heat shock and other chaperone proteins [25] that then help to guide proper folding of crucial cellular proteins at high temperature [26]. However, while there are myriad studies of HSR in the short term (i.e., on the scale of minutes), the sets of genes that contribute to *E. coli* thermal acclimation over the space of hours and days are not well known. Acclimation may prove to be a distinct physiological state, with specific energetic costs that merit further study. 

### Genetic associations and the effects of a large deletion

To assess whether phenotypic variation is driven by genetic variation that accrued during our adaptation experiment, we have associated genotypes with phenotypes. Our association analyses do not find a consequent phenotype for all of the mutational objects. For example, the gene *ybaL*, which was mutated in 65 of 115 lines, does not associate with any phenotypic axes. Nonetheless, we do find 117 genetic associations across eight PCA axes (Table 2), providing compelling evidence that at least some of the measured phenotypic variation has underlying genetic causations.

Among the many associations, the ECB_00503_large deletion is particularly surprising, because it is the major associate with the first two principal components of variation. In total, it associates with six of the nine principal components under study (Table 2), and it exhibits a strong signal of phenotypic differentiation when evolved clones are clustered hierarchically (Fig. 4). The ECB_00503_large deletion is also unique because it is the most common single mutation from the thermal evolution experiment; 35 of 115 evolved clones share this mutation. We previously speculated that the deletion has a high mutation rate due to homologous recombination between flanking IS insertions [5]. No matter the mutation rate, it is likely to have been under strong selection to reach high frequency in 35 independent populations.

**Fig. 5:**
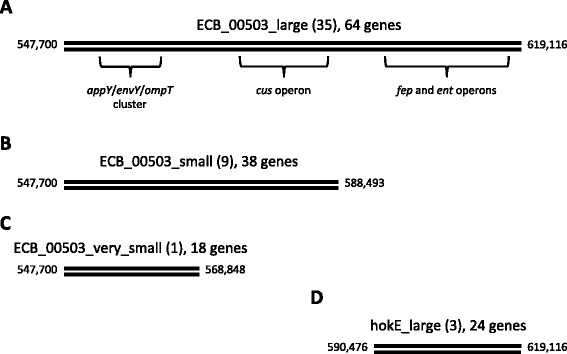
Schematic of the four overlapping deletion events found during the thermal evolution experiments. **a** The largest deletion (ECB_00503_large) removes 64 genes and was found in 35 of 115 evolved clones. **b** The smaller deletion (ECB_00503_small) was found in 9 of 115 clones and removed 38 genes. **c** An even smaller variant was found in 1 of 115 clones and removed 18 genes. **d** The hokE_large mutation removed 24 genes and was found in 3 of 115 clones. All variants are described in Tenaillon et al. [5]. In all diagrams, numbers at the 5′ and 3′ end of the schematic represent the base position on the reference genome [35]

The ECB_00503_large deletion is 71 kb in length and removes 64 genes (Fig. 5). These genes include the *cus* operon, which has been shown to be down-regulated in response to osmotic and heat stress [27], as well as the *fep* and *ent* operons, which regulate iron acquisition and are regulated by the iron-dependent master transcriptional regulator Fur [28]. Interestingly, Fur also regulates enzymes of glycolysis and the Krebs cycle, as well as enzymes that combat oxidative stress [29]. It seems possible that the deletion of iron acquisition genes and their Fur binding sites could, in theory, lead to pleiotropic effects by affecting the activation state of Fur or its titration on remaining binding sites. Single genes in the region could also play a role in the stress response, such as the transcription factors encoded by *appY* and *envY*, and the heat shock protease encoded by *ompT* (Fig. 5).

The ECB_00503_large deletion is one in a series of four overlapping deletions that permit preliminary dissection of the phenotypic effects of the *appY*/*envY*/*ompT* cluster, the *cus* operon, and the *fep*/*ent* operons (Fig. 5). For example, the hokE_large mutation removes the *fep*/*ent* operons, and this mutation associates with both pc2 and pc5 (Table 2). Hence, deletion of the *fep/ent* operon appears to be sufficient to generate some of the phenotypic variation caused by the larger deletion. Similarly, the ECB_00503_small mutation associates with pc4 (Table 2), suggesting that the region near *appY*/*envY*/*ompT* also contributes to phenotypic variation in our system.

Because the ECB_00503_large deletion has a large-effect on phenotype and also because deletions in this region were common among the complete set of 115 clones, we wondered whether the deletions could be solely responsible for the overall signal of restoration and convergence. To address this issue, we removed from our dataset all of the clones with deletions in the ECB_00503_large region and then estimated the proportion and direction of adaptation for the remaining 67 clones. We applied the same analytical procedures to this subset as to the entire dataset — i.e., we performed a PCA, assessed the number of significant eigenvectors (8 instead of 9 for the subset), performed *t*-tests on scores, counted the directionality of individual tests (Additional file 4: Table S2) and then calculated the proportion of variation attributable to each direction of adaptation. In this subset of clones, we find that full and partial restorations explain 44.7 and 14.8 % of observed phenotypic variation, respectively. Hence, the predominant directional signal is toward restoration of the pre-stress phenotype, even within clones that have not had a deletion in the ECB_00503_large region.

### Two adaptive trajectories: *rho* vs. *rpoB*

One of our motivating questions is whether the two adaptive trajectories defined by *rho* and *rpoB* lead to identical phenotypes and fitness optima. To that end, Rodriguez-Verdugo et al*.* [16] have documented that the two trajectories (as well as single mutations in the *rho* and *rpoB* genes) lead to different fitness trade-offs at low temperatures. Thus, the two trajectories do differ in phenotype in a low temperature environment. However, Rodriguez-Verdugo et al.(2014) were also not able to detect a difference in relative fitnesses between the two sets of clones at 42.2 °C, suggesting that the two trajectories may ascend ‘fitness peaks’ of similar height under thermal stress.

To better understand differences between the two trajectories, we applied MANOVA to our phenotypic data. The analyses revealed significant overall differences between the *rho* and *rpoB* trajectories and also identified factors that contribute to the difference. Based on these factors, the two trajectories appear to differ most substantially in chemical sensitivity but also in other aspects (Table 3).

Unfortunately, we cannot at this point infer the molecular causes of these phenotypic differences. We can, however, posit reasonable hypotheses. For example, the *rho* and *rpoB* trajectories differ in their associations with the *cls* gene; 23 of 30 clones with mutations in *rho* also contain a *cls* mutation, most of which interrupt *cls* function. In contrast, mutations within *cls* and *rpoB* are associated less often than expected by chance [5]; only 19 of 60 *rpoB* clones contain a *cls* mutation. These associations may be important because the *cls* gene produces a membrane lipid [30], and changes in membrane lipid composition are known to alter sensitivity to antibiotics and other chemicals [31, 32]. Hence, the two trajectories may differ in chemical sensitivity assays in part because of their different level of association with *cls* mutations. We note, however, that we have no insights as to why mutations within the *rho* and *cls* genes are statistically positively associated while mutations in *rpoB* and *cls* are not [5].

Another reasonable explanation for differences between the *rho* and *rpoB* trajectories is pleiotropy, because *rho* and *rpoB* mutations are expected to have different pleiotropic effects [16]. *rpoB* mutants have the capacity to affect the expression of every gene, but *rho* influences termination in only a subset of genes [33, 34]. Even if the two trajectories do differ in pleiotropic effects, the phenotypic differences we have documented here may not affect fitness under the conditions of the initial thermal stress experiment. However, they are likely to have consequential fitness effects in other environments, such as has been shown at low temperature [16].

## Conclusions

Overall, our data reveal that phenotypes converged predominantly toward states like those of the unstressed ancestor during our evolution experiment. This observation supports previous studies, which also document that adaptation in laboratory experiments consists largely of restorations toward the wild-type, pre-stress phenotype. Either plasticity or adaptation could drive phenotypic shifts, but phenotype-genotype associations confirm that at least some of the phenotypic change has a genetic component. In contrast to restoration, phenotypic novelty was less common but did explain as much as ~18 % of phenotypic variation. It remains an open question whether such novelty is merely a pleiotropic side effect of restorative evolution, or whether it provides some adaptive function of its own. Finally, our contrast of the *rho* and *rpoB* adaptive trajectories shows that the two represent different phenotypic spaces, but the interpretation of their effects is complicated by the compounded effects of several overlapping deletions as well as genetic mutations associated with each trajectory.

## Availability of supporting data

The PV values from the biolog assays (PV_Data_Table.xlsx), as well as the raw spectrophotometric measurements (Raw_Data_Table.xlsx), are available on the LabArchives database: dx.doi.org/10.6070/H4Nv9G79. The genotyping data for the clones was published previously and is available as an excel file (Additional file 4: Table S2.xls): http://www.sciencemag.org/content/335/6067/457/suppl/DC1

## Additional files

Additional file 1: Figure S1. Correlations between tests on the Biolog plates. A) The Pearson correlation coefficient between individual tests across all measured clones. B) The corresponding *p*-value for correlation coefficients. For both graphs, the axes represent the 94 tests on the Biolog plates. (PDF 212 kb) Additional file 2: Figure S2. Scree plot of the percent of variation explained by each principal component. The line corresponds to significance level, as determined by a bootstrapping heuristic. (PDF 5 kb) Additional file 3: Table S1. The loadings for each Biolog test in the first nine principal component. (XLS 58 kb) Additional file 4: Table S2. The number of directional comparisons in each principal component, along with the percent variation explained by each component. (DOCX 114 kb) Additional file 5: Figure S3. Q-Q plots for the results of association analyses. Each line represents one of the nine principal components. The diagonal represents the line for which there is no enrichment for tests with low *p-*values and therefore no evident biological signal. (PDF 157 kb)

## Abbreviations

PV: Phenotypic value
S: Score
OS: Optical density
