# Supplementary figures and images for: The phenotypic signature of adaptation to thermal stress in Escherichia coli
Source: BMC Evol Biol. 2015 Sep 2;15:177. doi: 10.1186/s12862-015-0457-3 (PMC4557228; doi:10.1186/s12862-015-0457-3)

A.

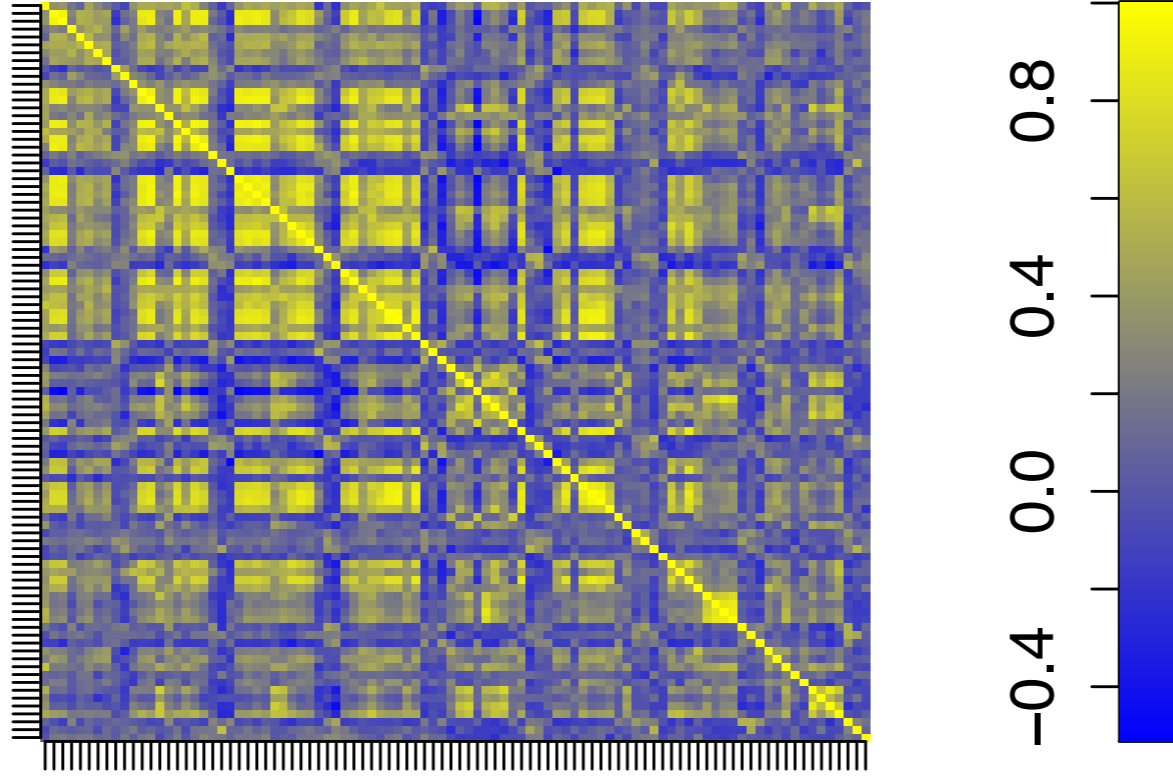

B.

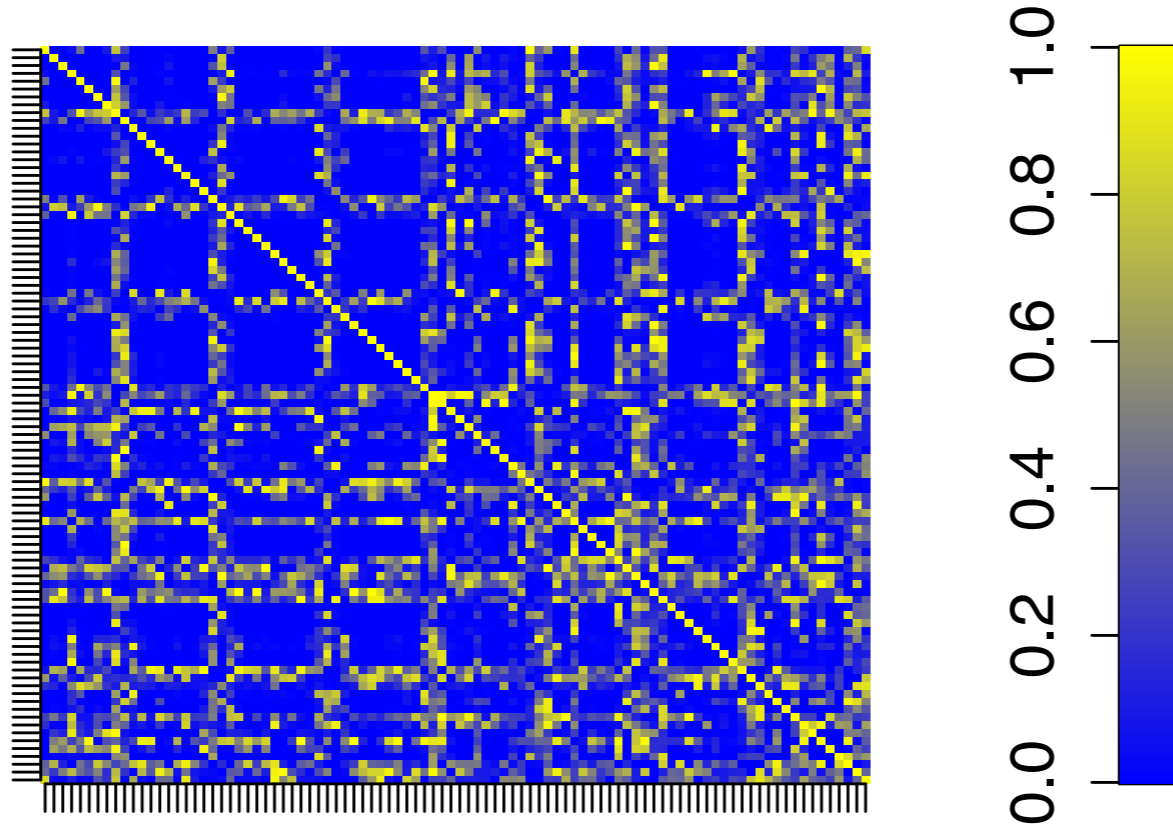

Supplement: Additional file 1: Figure S1. — Correlations between tests on the Biolog plates. A) The Pearson correlation coefficient between individual tests across all measured clones. B) The corresponding p-value for correlation coefficients. For both graphs, the axes represent the 94 tests on the Biolog plates. (PDF 212 kb) [file 12862_2015_457_MOESM1_ESM.pdf]

# Variance Explained by Each Principal Component

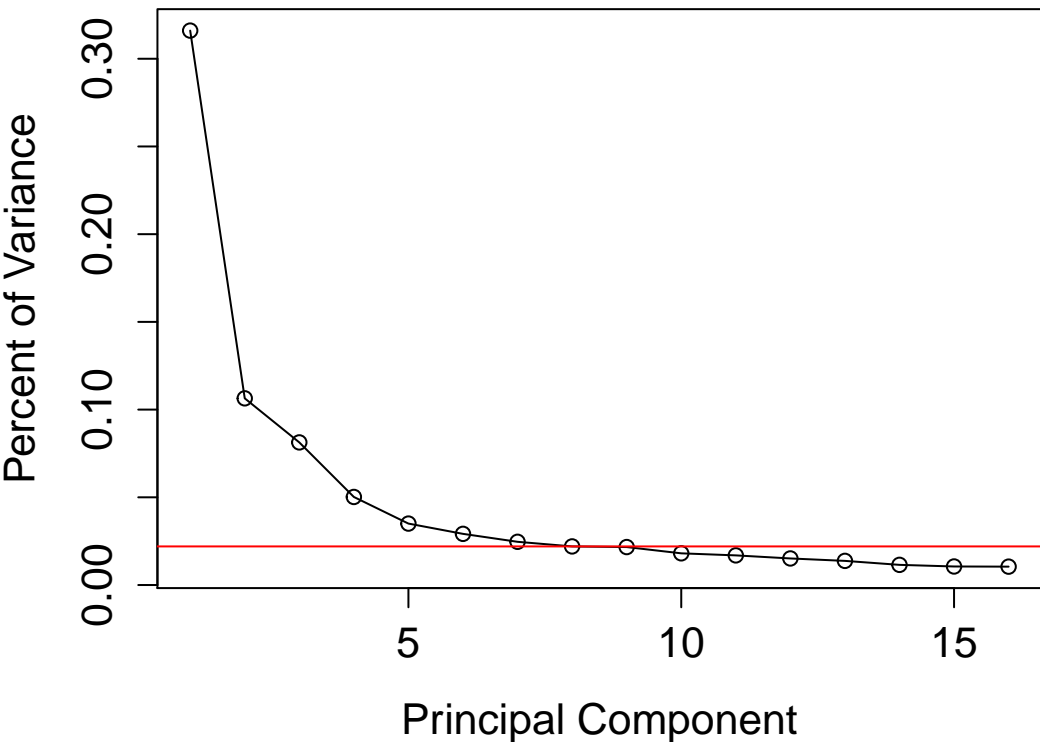

Supplement: Additional file 2: Figure S2. — Scree plot of the percent of variation explained by each principal component. The line corresponds to significance level, as determined by a bootstrapping heuristic. (PDF 5 kb) [file 12862_2015_457_MOESM2_ESM.pdf]

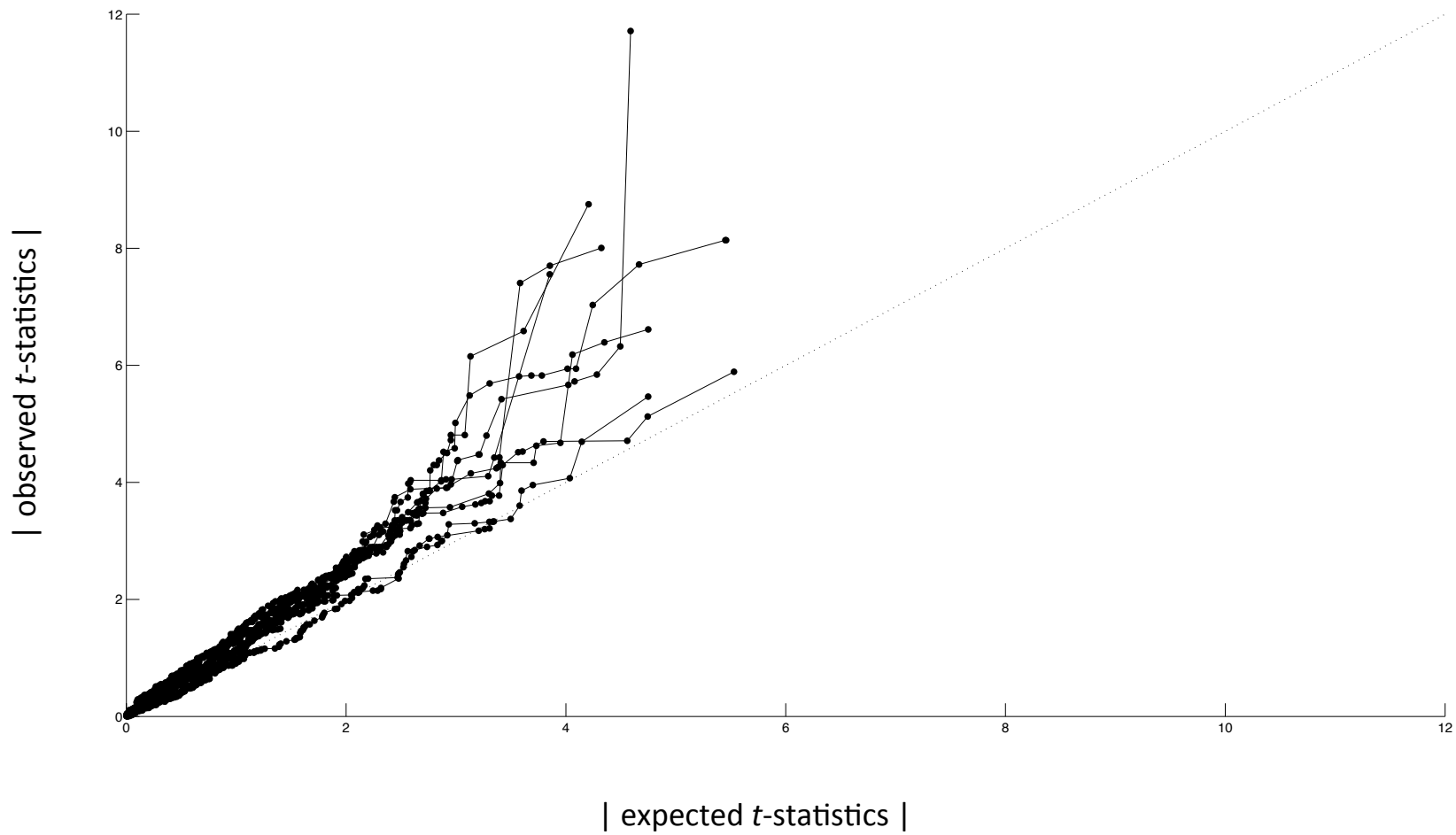

Supplement: Additional file 5: Figure S3. — Q-Q plots for the results of association analyses. Each line represents one of the nine principal components. The diagonal represents the line for which there is no enrichment for tests with low p-values and therefore no evident biological signal. (PDF 157 kb) [file 12862_2015_457_MOESM5_ESM.pdf]
