# Supplementary material for: The phenotypic signature of adaptation to thermal stress in Escherichia coli
Source: BMC Evol Biol. 2015 Sep 2;15:177. doi: 10.1186/s12862-015-0457-3 (PMC4557228; doi:10.1186/s12862-015-0457-3)
Supplement: Additional file 4: Table S2. — The number of directional comparisons in each principal component, along with the percent variation explained by each component. (DOCX 114 kb) [file 12862_2015_457_MOESM4_ESM.docx]

**Table S2**: The number and type of directional comparisons found in each principal component (pc), along with the proportion of phenotypic variance (PV%) captured by each axis.

| **Category^1^** | **pc1** | **pc2** | **pc3** | **pc4** | **pc5** | **pc6** | **pc7** | **pc8** | **pc9** |
| --- | --- | --- | --- | --- | --- | --- | --- | --- | --- |
|  | The full dataset of 115 clones | | | | | | | | |
| Partial | 49 | 8 | 0 | 0 | 18 | 18 | 31 | 0 | 31 |
| Unrestored | 6 | 52 | 0 | 0 | 76 | 53 | 32 | 1 | 20 |
| Restored | 28 | 4 | 0 | 0 | 0 | 3 | 6 | 51 | 31 |
| Reinforced | 0 | 44 | 0 | 0 | 20 | 5 | 7 | 0 | 3 |
| Inconsistent | 1 | 0 | 0 | 0 | 0 | 4 | 2 | 0 | 4 |
| Uninformative | 29 | 7 | 103 | 64 | 0 | 32 | 33 | 52 | 14 |
| Novel | 0 | 0 | 12 | 51 | 0 | 0 | 0 | 0 | 0 |
| Over | 2 | 0 | 0 | 0 | 1 | 0 | 4 | 11 | 12 |
| PV% | 0.3160 | 0.1064 | 0.0813 | 0.0502 | 0.03503 | 0.02917 | 0.02464 | 0.02201 | 0.02164 |
|  | The dataset of 67 clones without a deletion overlapping ECB_00503_large | | | | | | | | |
| Partial | 36 | 4 | 0 | 0 | 3 | 13 | 0 | 51 | NS^1^ |
| Unrestored | 5 | 23 | 0 | 0 | 26 | 2 | 36 | 7 | NS |
| Restored | 11 | 1 | 0 | 0 | 2 | 26 | 0 | 0 | NS |
| Reinforced | 0 | 12 | 0 | 0 | 31 | 2 | 7 | 0 | NS |
| Inconsistent | 0 | 0 | 0 | 0 | 0 | 0 | 0 | 0 | NS |
| Uninformative | 14 | 27 | 53 | 10 | 5 | 12 | 20 | 1 | NS |
| Novel | 0 | 0 | 14 | 57 | 0 | 0 | 0 | 0 | NS |
| Over-restored | 1 | 0 | 0 | 0 | 0 | 12 | 4 | 8 | NS |
| PV% | 0.3486 | 0.08642 | 0.08228 | 0.04804 | 0.03593 | 0.03426 | 0.02907 | 0.02503 | NS |

^1^ The directional categories are described Table 1 and Figure 1.

^2^ NS = not significant. With the reduced data set, only the first 8 principal components had a significant eigenvector, and so the ninth component was not considered.
